# Pediatric otogenic cerebral venous sinus thrombosis: a case report and a literature review

**DOI:** 10.1186/s13052-020-00882-9

**Published:** 2020-09-03

**Authors:** Massimo Luca Castellazzi, Giada Maria di Pietro, Michele Gaffuri, Sara Torretta, Giorgio Conte, Francesco Folino, Sebastiano Aleo, Samantha Bosis, Paola Marchisio

**Affiliations:** 1 Fondazione IRCCS Ca’ Granda Ospedale Maggiore Policlinico, Paediatric Emergency Department, Milan, Italy; 2 University of Milan, Milan, Italy; 3 Fondazione IRCCS Ca’ Granda Ospedale Maggiore Policlinico, Department of Otolaryngology and Head and Neck Surgery and Department of Clinical Sciences and Community Health, University of Milan, Milan, Italy; 4 Fondazione IRCCS Ca’ Granda Ospedale Maggiore Policlinico, Neuroradiology Unit, Milan, Italy; 5 Fondazione IRCCS Ca’ Granda Ospedale Maggiore Policlinico, Paediatric Highly Intensive Care Unit, Milan, Italy; 6 Department of Pathophysiology and Transplantation, University of Milan, Milan, Italy

**Keywords:** Acute otitis media, Acute mastoiditis, Cerebral venous sinus thrombosis, *Fusobacterium necrophorum*, Children

## Abstract

**Background:**

Cerebral venous sinus thrombosis in children is a rare but potentially fatal complication of acute mastoiditis, one of the most common pediatric infectious diseases. Due to its subtle clinical presentation, suspicion is essential for a prompt diagnosis and appropriate management. Unfortunately, no standard treatment options are available. To discuss the possible clinical presentation, microbiology, and management, we here report the case of a child with otogenic cerebral venous sinus thrombosis and perform a literature review starting from 2011.

**Case presentation:**

The child, a 10-months-old male, presented clinical signs of right acute otitis media and mastoiditis. Brain computed tomography scan detected right sigmoid and transverse sinus thrombosis, as well as a subperiosteal abscess. *Fusobacterium necrophorum* and *Haemophilus Influentiae* were detected on cultural sampling. A multidisciplinary approach along with a combination of medical and surgical therapy allowed the patient’s full recovery.

**Conclusion:**

Cerebral venous sinus thrombosis is a rare but severe complication of acute otitis media and mastoiditis. The management of this pathological condition is always challenging and an interdisciplinary approach is frequently required. Current therapeutic options include a combination of medical and surgical therapy. A patient-centered approach should guide timing and treatment management.

## Background

Acute otitis media (AOM) is one of the most common infectious diseases in the pediatric age group, with at least 60% of children under the age of 3 having experienced at least one episode, and approximately 24% three or more episodes [1].

The most frequent AOM complication is acute mastoiditis (AM); more severe complications such as facial paralysis, meningitis, subperiosteal, epidural, or intracerebral are still possible albeit rare [2].

Cerebral venous sinus thrombosis (CVST) is a possible severe complication of AM, with an estimated incidence rate of 0–2.7% [3]. It has been associated with neurological sequelae and is potentially fatal if not promptly diagnosed and treated [4]. Specific management of the condition, however, is still a matter of debate [5, 6].

## Case presentation

**Fig. 1:**
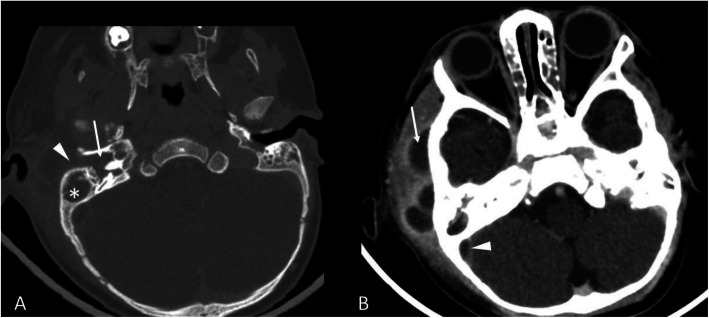
**a** On bone window of the pre-contrast CT showed complete obliteration of the tympanic cavity (arrow), the mastoid (asterisk) and the external auditory canal (arrowhead), compatible with an otomastoiditis. **b** Contrast-enhanced CT showed multiple abscesses in the right peri-auricular soft-tissue (arrow) and the thrombosis of the right sigmoid sinus (arrowhead)

A previously healthy 10-months-old patient was admitted to the pediatric emergency department of our hospital with a two-day long fever, irritability and right otorrhea, which worsened a few hours before admission. Clinical examination showed right otorrhea associated with eversion of the auricular pinna, retroauricular swelling, skin redness, tenderness, and pain on palpation of the mastoid region. No signs of neurological impairment or meningeal involvement were detected. Laboratory tests revealed a white blood cell count of 11,860/mmc with neutrophil predominance, and an elevated C-reactive protein (CRP) of 15,76 mg/dl (normal value < 0.5 mg/dL). A blood bacterial culture was also performed and resulted negative. The patient was immediately started on ceftriaxone (100 mg/kg/day), however, worsening of the local clinical objectivity the next day prompted its replacement with a combination of meropenem (100 mg/kg/day) and vancomycin (40 mg/kg/day). A contrast-enhanced (CE) computed tomography (CT) scan of the head showed bilateral mastoiditis with swelling of the adjacent right soft tissues, multiple abscesses, and a thrombosis of the right sigmoid sinus and of the distal portion of the right transverse sinus (Fig. 1). The patient underwent a right canal wall up (CWU) mastoidectomy, with skeletonization of the cortical bone for sinus management, and a right myringotomy with placement of a ventilation tube.

The bacterial culture of the purulent drainage was positive for *Fusobacterium (F.) necrophorum* and *Haemophilus (H.) Influentiae* sensitive to all antibiotics tested.

Treatment of the sinus thrombosis was initiated on the day after surgery with subcutaneous low molecular weight heparin (LMWH) was administered at the standard dosage of 100 International Units (IU)/kg twice a day, to treat the sinus thrombosis. Ten days later, LMWH dosage was reduced to 70 IU/kg twice a day following the detection of a prolonged activated partial thromboplastin time (aPTT) ratio of 1,66 (normal value 0,86-1,2), and suboptimal serum anti-factor Xa levels.

**Fig. 2:**
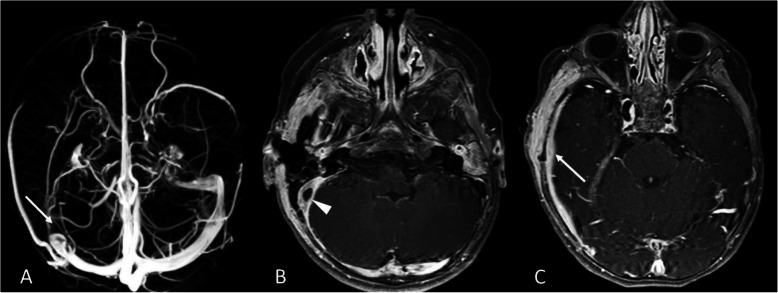
**a** Magnetic Resonance Venography showed the absence of the flow-related signal in the right sigmoid sinus (arrow). **b** The contrast-enhanced T1-weighted sequence showed the thrombosis and the wall-thickening of the right sigmoid sinus (arrowhead), as well as (**c**) the inflammatory thickening and the contrast-enhancement of dura mater (arrow) in the right temporal region

Three days after surgery, fever persistence prompted the execution of a contrast-enhanced magnetic resonance imaging (MRI) of the head which confirmed right sigmoid sinus thrombosis, inflammation of the soft tissues behind the right ear, and pachymeningitis of the right temporal region (Fig. 2).

The antibiotic treatment with meropenem and vancomycin was continued for a total of 6 weeks with a progressive resolution of the fever (the patient was apyretic after 10 days of antibiotic treatment), and of the general and local conditions. A gradual normalization of the white blood cell count and CRP were also documented. A bacterial culture test on the exudate performed 2 weeks after surgery was negative.

Immunological tests including immunoglobulins and IgG subclasses, lymphocyte subpopulations, tests for complement function (CH50, AP50) were also performed, but returned no significative result. Abdominal ultrasound and cardiologic examination were normal.

The patient was discharged in good general conditions after 6 weeks of hospitalization and underwent prolonged anticoagulation therapy for 6 months.

A head MRI performed 4 months later revealed a partial recanalization of the right transverse and sigmoid sinus, a mild improvement in the appearance of the right mastoid inflammation, and an enhancement of the right preauricular soft tissues.

After 6 months, the MRI showed a complete resolution of the mastoiditis with no soft tissue involvement and a further improvement of the venous sinus thrombosis.

## Discussion and conclusion

Otogenic CVST is a rare condition in the pediatric age group, but has a high mortality rate (5–10%) and can be associated with severe clinical morbidities if not promptly diagnosed and treated [7]. How the disease should be managed, however, is still a matter of debate. To better discuss possible clinical presentation, pathogenesis, diagnosis, and treatment, we performed a literature review of pediatric cases of otogenic CVST published since 2011. Several such studies have been published, and Table 1 summarizes their main features. Different factors contribute to the development of an otogenic CVST. The proximity of the sigmoid sinus to the mastoid, for example, allows adjacent inflammation to activate platelets and fibrin possibly resulting in a mural thrombus [25]. Subsequently, this thrombus may extend to the adjacent dural venous sinuses (transverse, inferior, or superior petrosal) and to the internal jugular vein (IJV). A dural venous sinus thrombosis may also determine a reduced reabsorption of cerebrospinal fluid which may determine an increased intracranial pressure favoring a condition known as otitic hydrocephalus [22].

**Table 1:** Summary of the reported cases of otogenic sinus thrombosis in children from 2011 to 2020

Author, year, nation, reference	N. of patient	Age	Gender (M/F)	Onset symptoms	Neurological complication	Sinus involved	Intracranial complication	Surgical treatment	Anticoagulation (Duration)	Antibiotic Treatment	Clinical Outcome	Radiological Outcome
Bevan, 2020, United Kingdom [8]	11	3–11 years	7 M 4 F	Not reported	Papilledema, Abducens nerve palsy, strabismus, visual defect, seizures	2 SS 2 TS 4 SS + TS + IJV 1 SS + IJV 1 SS + TS 1 IJV	1 epidural abscess 11 otitic hydrocephalus	7mastoidectomy 3 mastoidectomy and myringotomy 1 mastoidectomy, myringotomy, craniotomy	LMWH; Rivoxaban (not clarified)	Broad spectrum antibiotic	Not clarified	Not clarified
Coutinho, 2018, Portugal [6]	16	2–16 years	7 M 9 F	Ear pain, headache, lethargy, nausea, vomiting, fever neck stiffness	Abducens nerve palsy, papilledema	4 SS 2 TS 6 SS, TS 4 SS, TS, IJV	5 epidural abscess 3 otitic hydrocephalus 1 epidural abscess, meningitis	16 Mastoidectomy, transtimpanic ventilation tube 7 perioperative sigmoid sinus exposure 3 drainage perisinus empyema 5 craniotomy	UFH; LMWH; warfarin (3–12 months)	Broad spectrum antibiotic	2 neuro-ophtalmological impairment 1 HDAD 1 non-specific behaviour disorder	3 partial recanalization 7 complete recanalization
Scorpecci, 2018, Italy [9]	25	1–14 years	17 M 8 F	Ear pain,signs of mastoiditis, headache, othorrea, neck stiffness	VI cranial nerve palsy, altered consciousness, papilledema, vertigo	16 SS 4 SS + TS 1 SS + cavernous sinus 1 SS + sagittal sinus 2 SS + jugular bulb	2 epidural abscess 1 cerebellar abscess	16 mastoidectomy and tympanostomy tube insertion 3 abscess evacuation through an occipital craniotomy approach	LMWH (at least 2 months)	Broad spectrum antibiotic	1 persistent bilateral optic nerve atrophy and subsequent impaired visual acuity	20 complete recanalization 2 partial recanalization 3 no complete follow-up
Scherer, 2017, USA [10]	1	6 years	M	Headache, ear pain, blurry vision, nausea, vomiting	Papilledema, Abducens nerve palsy	SS, TS, IJV	None	Mastoidectomy, myringotomy, tube placement	LMWH (6 months)	Broad spectrum antibiotic	No sequelae	Complete recanalization
Ryan, 2016, USA [11]	7	6–15 years	4 M 3 F	Fever, ear pain, othorrea, neck stiffness	Papilledema	7 SS 7 TS 5 IJV	2 epidural abscess 2 otitic hydrocephalus 1 meningitis 1 cavernous sinus thrombosis	Mastoidectomy and tympanostomy tube 2 epidural abscess drainage	5 patients; not clarified	Broad spectrum antibiotic	1 right visual-field deficit 6 no sequelae	In 1 patient persistent thrombosis after 9 months. Not clarified for the other patients
Zanoletti, 2015, Italy [7]	8	2–7 years	4 M 4F	Fever, headache, ear pain lethargy	6 papilledema 3 diplopia 2 photophobia 1 facial paralisis	8 SS 6 TS 4 IJV	1 epidural abscess	5 mastoidectomy 2 transtimpanic drainage 2 myringocentesis	UFH; LMWH (3 months)	Broad spectrum antibiotic	No sequelae	5 complete recanalization 1 partial recanalization 1 sinus entirely distrupted
Rosdy, 2014, Hungary [12]	10	4–8 years	6 M 4 F	Fever, headache, ear pain, otorrhea, lethargy, vomiting, neck stiffness, torcicollis	9 papilledema 2 abducens nerve palsy 3 ataxia	10 SS 5 TS 3 IJV	4 perisinous abscess 1 epidural abscess 1 cerebellar abscess	10 mastoidectomy 2 thrombectomy 1 IJV ligation 1 epidural abscess drainage	8 patients LMWH (3–6 months months)	Broad spectrum antibiotic	1 unilateral visual deficit	5 complete recanalization 2 partial recanalization
Funamura, 2014, USA [5]	5	1–15 years	4 M 1 F	Fever, ear pain, headache, nausea, vomiting, otorrhea, lethargy	1 patient presented seizures after intracranial hemorrage	5 SS 4 TS 2 IJV	2 epidural abscess 1 brain abscess	4 mastoidectomy 1 myringotomy with tympanostomy tube 1 IJV drainage 1 craniotomy with drainage of brain abscess 3 epidural abscess drainage	3 patients UFH (3 days-3 weeks) followed by LMWH (3–6 months)	Broad spectrum antibiotic	1 epilepsy and developmental delay 1 headache	2 partial recanalization 1 complete recanalization
Ulanovski, 2014, Israel [13]	24	7 months-12 years	10 M 14 F	Fever, ear pain	1 abducens nerve palsy 3 seizures	9 SS 6 SS + TS 4 SS + IJV 4 SS + TS + IJV 1 cavernous sinus	11 epidural involvement 5 temporal bone osteomyelitis	21 mastoidectomy + decompression of the sinus 3 none intervention	22 patients LMWH (3–6 months)	Broad spectrum antibiotic	No sequelae	7 complete recanalization 6 partial recanalization 2 persistence obstructed vessels
Au, 2013, USA [14]	1	14 years	M	Fever, ear pain, otorrhea, headache, vomiting, fatigue, visual allucinations, blurry vision	Not reported	TS + SS	Epidural abscess	Myringotomy, tube placement, mastoidectomy	Not performed	Broad spectrum antibiotic	No sequelae	Complete recanalization
Novoa, 2013, Switzerland [15]	9	1–13 years	6 M 3 F	Fever, ear pain, otorrhea, headache, vomiting, apathy	2 abducens nerve palsy 1 vertigo	5 SS 3 SS+ IJV 1 SS + TS	4 otitis hydorcephalus 4 subperiostal abscess 2 epidural abscess	Mastoidectomy and tympanostomy tubes	6 patients LMWH (3–6 months) 3 patients UFH (2,7,21 days) followed by LMWH in 1 patient (3 months), acetylsalicylate in 1 patient (6 months), phenprocoumon in 1 patient (11 months)	Broad spectrum antibiotic	1 patient presented moderate hearing loss, persistence of increased intracranial pressure, headache, legasthenia and atrophy of optic nerve	7 complete recanalization 2 persistent recanalization
Csakanyi, 2013, Hungary [16]	8	4–8 years	6 M 2 F	Fever, ear pain, headache, lethargy, nausea, vomiting, neck stiffness, torcicollis	1 ataxia 1 bradycardia	2 SS 1 SS + TS 4 SS + TS + IJV 1 only granulation	Not reported	6 mastoidectomy + decompression of sinus 1 mastoidectomy + thrombectomy 1 mastoidectomy + thrombectomy + IJV ligation	6 patients LMWH (2–6 months)	Broad spectrum antibiotic	1 permanent visual loss	7 complete recanalization In patient with IJV involvement a good collateral circulation was observed
Van Munster, 2013, The Netherlands [17]	1	3 years	F	Fever, left side otorrhea, vomiting	abducens nerve palsy	Sinus thrombosis (not specified)	Cerebellar empyema	Mastoidectomy + thrombectomy	LMWH (not clarified)	Broad spectrum antibiotic	No sequelae	Complete recanalization
Inkuchi, 2013, Japan [18]	1	5 years	M	Nausea, vomiting, headache	None	TS + SS + IJV	Not reported	Ventricle-peritoneal shunt + ventilation tube insertion	Not performed	Not reported	No sequelae	Complete recanalization
Sitton, 2012, United States [19]	7	2–15 years	5 M 2 F	Fever, ear pain, otorrhea, vomiting, headache, neck stiffness, mastoid tenderness	Diplopia	SS (2) SS + TS (3) SS + IJV (2)	1 subperiostal abscess	3 mastoidectomy + myringotomy and tube placement 1 mastoidectomy + aspiration of sinus 1 myringotomy, tube placement and drainage of subperiostal abscess 2 myringotomy, tube placement	6 patients LMWH. Of these 3 patients previously received UFH and 4 switched to warfarin. (1.5–6 months)	Broad spectrum antibiotic	No sequelae	6 complete recanalization 1 no resolution
Zangari, 2012, Italy [20]	5	3–10 years	4 M 1 F	Fever, headache, asthenia, vomiting, signs of mastoiditis	Diplopia, photophobia	3 SS 2 SS + TS	None	2 mastoidectomy 3 none	LMWH for 3 months. In 1 patient LMWH was followed by oral anticoagulant for 6 months.	Broad spectrum antibiotic	No sequelae	3 complete recanalization 1 partial recanalization 1 no resolution
Ropposch, 2012, Austria [21]	6	3–15 years	6 M	Headache, neck stiffness, fever, ear pain, post-auricular pain and erythema, otorrhea	1 Vertigo 1 Abducens nerve palsy	3 SS + TS 3 SS + IJV	None	3 mastoidectomy + thrombectomy 2 mastoidectomy + ligation IJV	UFH followed by LMWH (3 months)	Broad spectrum antibiotic	1 hydrocephalus and papilledema that rgressed after 6 months	3 complete recanalization
Ghosh, 2011, USA [22]	13	5 months-18 years	9 M 4 F	Headache, vomiting, fever, otorrhea	4 abducens nerve palsy 1 facial nerve palsy 5 papilledema Ataxia	8 TS 5 TS + SS + IJV	None	4 mastoidectomy 5 mastoidectomy + myringotomy and ventilation tube 1 myringotomy with tube ventilation	3 LMWH (6 months)	Broad spectrum antibiotic	4 transient hearing loss 1 permanent hearing loss	3 complete recanalization 2 partial recanalization
Bielecki, 2011, Poland [23]	5	3–9 years	3 M 2 F	Headache, ear pain, fever, vomiting	6 papilledema 3 diplopia 3 facial nerve palsy	1 SS 1 SS + TS 1 SS + IJV 1 SS + TS + IJV 1 SS + TS + IJV + SGS	None	5 mastoidectomy + ventilation tube placement	4 UFH, followed by LMWH and acenocoumarol (6 months)	Broad spectrum antibiotic	No sequelae	5 partial or complete recanalization (not clarified)
Viswanatha, 2011, India [24]	9	8–12 years	6 M 3 F	Headache, ear pain, fever, vomiting, vertigo	1 Facial nerve palsy and lateral rectus palsy	TS	4 cerebellar abscess 3 meningitis, 1 temporal lobe abscess 1 otitic hydrocephalus	9 mastoidectomy + thrombectomy drainage of intracranial abscesses	Not performed	Broad spectrum antibiotic	No sequelae	Not reported
Neilan, 2011, USA [25]	15	6 months - 14 years	10 M 5 F	Not reported	5 diplopia	2 SS 3 SS + TS 1 SS + IJV 8 SS + TS + IJV 1 SS + CS + IJV	5 otitic hydrocephalus	15 mastoidectomy and tympanostomy tube placement + 3 needle decompression 3 osseous decompression 6 venotomy	12 patients LMWH (6 weeks-6 months)	Not reported	Not reported	4 persistence obstructed vessels 2 complete recanalization 8 partial recanalization

*SS* Sigmoid sinus, *TS* Transverse sinus, *IJV* Internal jugular vein, *SGS* Sagittal sinus, *UFH* Unfractionated heparin, *LMWH* Low-molecular-weight heparin, *HDAD* Hyperactivity disorder and attention deficit

The classical signs and symptoms of otogenic CVST following AM are high-grade “picket fence” fever, otalgia, otorrhea, and altered mental status [14]. However, the use of antibiotics for AOM may result in a more insidious presentation. Results from our literature review show that fever is one of the most frequent clinical signs upon presentation [5–7, 11–17, 19–21, 23, 24], followed by headache [5–7, 9, 10, 12, 14–16, 18–24], ear pain and/or otorrhea [5–7, 9–17, 19–24], nausea and/or vomiting [5, 6, 10, 12, 14–18, 22–24], lethargy [5–7, 12, 16], neck stiffness [9, 11, 12, 16, 19, 21], and signs of mastoiditis [9, 19–21]. The most common neurologic signs at presentation of otogenic CVST in children were found to be: papilledema [6–12, 22, 23], abducens nerve palsy [6, 8–10, 12, 13, 15, 17, 21, 22], diplopia [7, 19, 20, 23, 25], facial nerve palsy [7, 22–24], seizures [5, 8, 13], ataxia [12, 16, 22], vertigo [9, 15, 21], and strabismus [8]. Patients presenting with these signs and symptoms should undergo imaging to exclude or confirm otogenic CVST. A CT scan may be used, although MRI, magnetic resonance venography, and angiography with venous phase should be preferred for diagnosing otogenic CVST and its complications [14]. Intracranial complications that must be excluded are otitic hydrocephalus [6, 8, 11, 15, 24, 25], epidural abscess [5–8, 11–15], intracranial abscess [5, 12, 15, 17, 19, 24], and meningitis [6, 11, 24]. Use of MRI should be considered not only for diagnosis, but also for the follow-up of these patients, as it could potentially reduce the exposure to high doses of ionizing radiation [14].

From a microbiological perspective, most cases of pediatric otogenic CVST have negative bacterial culture tests. When positive the most common isolated bacteria are represented by *Streptococcus pyogenes*, *Streptococcuspneumoniae*, *Staphylococcus aureus*, *H. influentiae,* and *Pseudomonas aeruginosa* (for more details see Table 2).

**Table 2:** The main pathogens associated with otogenic CVST in children

Pathogen	Reference
No growth	[5–7, 10–13, 15, 21–25]
*Streptococcus pneumoniae*	[11–13, 21–23, 25]
*Streptococcus pyogenes*	[6, 7, 11, 14, 21, 25]
*Pseudomonas aeruginosa*	[6, 22, 24]
*Proteus mirabilis*	[6, 11, 22]
*Staphylococcus aureus*	[6, 13, 25]
Fusobacterium necrophorum	[13, 17]
Haemophilus influentiae	[5, 13]
Other pathogens	[5, 6, 11, 13, 15, 21, 22, 24, 25]
Not reported	[8, 9, 16, 18–20]

In our case, bacterial cultures performed during surgery identified *H. influentiae* and *F. necrophorum.* The latter has been identified in other 3 cases and seems to be associated with a more aggressive disease course, and osteomyelitis [13, 17]. This is in line with our clinical findings. *F. necrophorum* is a Gram-negative anaerobic bacillus, which is known to be part of the microbiome of the oral cavity, gastrointestinal tract, and female genital tract [26]. It is responsible for a wide range of severe infections of the head and neck such as peritonsillar abscesses and mastoiditis [27]. A significant association with otogenic CVST (*P* < .001) was first observed in a recent retrospective study by Coudert et al. When compared to the CVST from other bacteria groups, the same study showed that children in the CVST *Fusobacterium* group were significantly younger (61 months vs 23 months, *P* < .01) and had a more severe clinical presentation, with a higher CRP and larger subperiosteal abscess’. These patients generally required a combination of medical and surgical treatment and a longer hospital stay [28].

Once otogenic CVST is diagnosed, empiric antibiotic therapy should be initiated. If a specific pathogen is later identified, more specific antimicrobial agents should replace the initial treatment [14]. For how long the antibiotic treatment should be continued is still uncertain. In consideration of the more aggressive clinical presentation, a one-month antibiotic course has been suggested for *Fusobacterium* infections [28].

Anticoagulation therapy and surgical treatment in otogenic CVST remain areas of debate.

Anticoagulation may be useful in restricting the thrombus’ extension, in promoting intracranial drainage, and thus in limiting a rise in intracranial pressure [29]. Anticoagulation, however, may be associated with severe complications such as bleeding, drug interaction, thrombocytopenia, osteoporosis, and hemorrhagic skin necrosis [19].

Recent guidelines recommend treating children affected by CVST with LMWH [30, 31]. However, different studies still give different anticoagulation approaches in terms of treatment duration and of which anticoagulant to use. We opted for a LMWH in the standard dosage of 100 IU/kg twice a day, which was then reduced to 70 IU/kg twice a day when a prolonged aPTT ratio and suboptimal serum anti-factor Xa levels were detected. The patient was administered LMWH for a total of 6 months. This anticoagulation regimen is similar to that proposed in a recent retrospective study by Scorpecci et al. [9]. The authors suggested that anticoagulation therapy with LMWH should be started immediately after diagnosis and continued for 2 months or longer in those patients who do not achieve recanalization or in those who present a high-risk thrombophilia. Moreover, the authors proposed that all patients with an otogenic CVST diagnosis should be screened for thrombophilia in order to evaluate the risk of thrombosis recurrence and treatment duration [9, 32]. Nonetheless, thrombophilia screening remains a matter of debate as it is expensive and no evidence of robust proof of its relevance exists [28].

From a surgical point of view, the current trend is to perform a mastoidectomy with the removal of inflammatory tissue from the sinus’ walls, in order to obtain the eradication of the perisinus infection [7, 33, 34]. To promote both drainage and aeration of the middle ear, aditus ad antrum, and mastoid antrum, and thus compensate for the pressure exerted from the purulent effusion, the mastoidectomy can be carried out in association with a myringotomy, with or without tube placement [6]. More aggressive options such as surgical sinus drainage with removal of the thrombus are not routinely recommended [11, 19]. IJV ligation is limited to cases with persistent septicemia or septic pulmonary emboli [5]. We opted for a CWU mastoidectomy with drainage of the subperiosteal abscess, myringotomy, and placement of a ventilation tube.

In conclusion, pediatricians should be aware of this severe and potentially lethal complication of AM, especially those cases with a *F. necrophorum* infection. Although there is still no unanimous agreement on what treatment is best for these patients, a prompt diagnosis is essential for appropriate management and a good outcome.

## Data Availability

Data sharing was not applicable to this case report because no datasets were generated or analysed during the study.

## Abbreviations

AOM: Acute otitis media
AM: Acute mastoiditis
CVST: Cerebral venous sinus thrombosis
CRP: C-reactive protein
CE: Contrast-enhanced
CT: Computed tomography
CWU: Canal wall up
F.: Fusobacterium
H.: Haemophilus
MRI: Magnetic resonance imaging
LMWH: Low molecular weight heparin
IU: International Units
aPTT: Activated partial thromboplastin time
